# Malic Enzyme, not Malate Dehydrogenase, Mainly Oxidizes Malate That Originates from the Tricarboxylic Acid Cycle in Cyanobacteria

**DOI:** 10.1128/mbio.02187-22

**Published:** 2022-10-31

**Authors:** Noriaki Katayama, Kaori Iwazumi, Hiromi Suzuki, Takashi Osanai, Shoki Ito

**Affiliations:** a School of Agriculture, Meiji University, Kawasaki, Kanagawa, Japan; University of California, Irvine

**Keywords:** aerobic respiration, cyanobacteria, malate dehydrogenase, malic enzyme, tricarboxylic acid cycle

## Abstract

Oxygenic photoautotrophic bacteria, cyanobacteria, have the tricarboxylic acid (TCA) cycle, and metabolite production using the cyanobacterial TCA cycle has been spotlighted recently. The unicellular cyanobacterium *Synechocystis* sp. strain PCC 6803 (*Synechocystis* 6803) has been used in various studies on the cyanobacterial TCA cycle. Malate oxidation in the TCA cycle is generally catalyzed by malate dehydrogenase (MDH). However, *Synechocystis* 6803 MDH (*Sy*MDH) is less active than MDHs from other organisms. Additionally, *Sy*MDH uses only NAD^+^ as a coenzyme, unlike other TCA cycle enzymes from *Synechocystis* 6803 that use NADP^+^. These results suggest that MDH rarely catalyzes malate oxidation in the cyanobacterial TCA cycle. Another enzyme catalyzing malate oxidation is malic enzyme (ME). We clarified which enzyme oxidizes malate that originates from the cyanobacterial TCA cycle using analyses focusing on ME and MDH. In contrast to *Sy*MDH, *Synechocystis* 6803 ME (*Sy*ME) showed high activity when NADP^+^ was used as a coenzyme. Unlike the *Synechocystis* 6803 mutant lacking *Sy*MDH, the mutant lacking *Sy*ME accumulated malate in the cells. ME was more highly preserved in the cyanobacterial genomes than MDH. These results indicate that ME mainly oxidizes malate that originates from the cyanobacterial TCA cycle (named the ME-dependent TCA cycle). The ME-dependent TCA cycle generates NADPH, not NADH. This is consistent with previous reports that NADPH is an electron carrier in the cyanobacterial respiratory chain. Our finding suggests the diversity of enzymes involved in the TCA cycle in the organisms, and analyses such as those performed in this study are necessary to determine the enzymes.

## INTRODUCTION

Cyanobacteria are prokaryotes that perform oxygenic photosynthesis. In recent years, during which global warming and marine pollution have become global issues, cyanobacteria have been identified as ideal hosts for eco-friendly and sustainable metabolite production from carbon dioxide (1, 2). The non-nitrogen-fixing unicellular cyanobacterium *Synechocystis* sp. strain PCC 6803 (*Synechocystis* 6803) has many characteristics that make it suitable as a model organism, such as natural transformation capability (3), whole-genomic information (4), and tolerance for cryopreservation. Therefore, *Synechocystis* 6803 has been widely used in both basic and applied studies.

The tricarboxylic acid (TCA) cycle is one of the most important metabolic pathways for the generation of energy and amino acids. Cyanobacteria do not possess a 2-oxoglutarate dehydrogenase complex that catalyzes the conversion of 2-oxoglutarate to succinyl coenzyme A (succinyl-CoA) in the TCA cycle (5, 6). Therefore, the cyanobacterial TCA cycle was thought for over 4 decades to be incomplete. However, in 2011, it was discovered that 2-oxoglutarate decarboxylase and succinic semialdehyde dehydrogenase catalyze the conversion of 2-oxoglutarate to succinate in the cyanobacterial TCA cycle (7). Since then, the cyanobacterial TCA cycle has been considered complete, and various studies regarding the cyanobacterial TCA cycle have been conducted. In *Synechocystis* 6803, the γ-aminobutyric acid shunt also contributes to the conversion of 2-oxoglutarate to succinate in the TCA cycle (8). The glyoxylate cycle, which is a variant of the TCA cycle, is important for acetate assimilation in some nitrogen-fixing cyanobacteria (9). In recent years, studies of metabolite production using the TCA cycle in *Synechocystis* 6803 have been well performed (10–14). However, in *Synechocystis* 6803, metabolic flux through the TCA cycle is lower than that through other carbon metabolic pathways, such as glycolysis (15). Understanding the biochemical properties of the cyanobacterial TCA cycle is necessary to improve metabolic flux through the TCA cycle. Until 2016, only isocitrate dehydrogenase was biochemically analyzed among the TCA cycle enzymes in *Synechocystis* 6803 (see Table S1 in the supplemental material) (16). Recently, biochemical analyses of TCA cycle enzymes in *Synechocystis* 6803 have been performed (Table S1) (17–22).

10.1128/mbio.02187-22.8 TABLE S1 List of the TCA cycle enzymes in *Synechocystis* 6803 that have been biochemically analyzed and not. Download Table S1, DOCX file, 0.02 MB. Copyright © 2022 Katayama et al. 2022 Katayama et al. https://creativecommons.org/licenses/by/4.0/ This content is distributed under the terms of the Creative Commons Attribution 4.0 International license.

Malate dehydrogenase (MDH; EC 1.1.1.37) catalyzes the following reversible redox reactions in the TCA cycle: malate + NAD^+^ ↔ oxaloacetate + NADH. *Synechocystis* 6803 MDH (*Sy*MDH) has lower malate oxidation activity than MDHs from other organisms and specifically catalyzes the reductive reaction (18). In addition, *Sy*MDH uses only NAD^+^ as a coenzyme and has no enzymatic activity for NADP^+^ (18), a coenzyme of the other two enzymes that catalyze the NAD(P)H-generating reaction in the TCA cycle in *Synechocystis* 6803 (isocitrate dehydrogenase and succinic semialdehyde dehydrogenase) (Table S1) (16, 21). These results suggest that MDH seldom catalyzes malate oxidation in the cyanobacterial TCA cycle, and the enzyme(s) that oxidizes malate that originates from the cyanobacterial TCA cycle remains unclear.

The other enzyme conserved in most organisms that can catalyze malate oxidation is malic enzyme (ME; EC 1.1.1.38, 1.1.1.39, 1.1.1.40). ME catalyzes the following reversible redox reaction: malate + NAD(P)^+^ ↔ pyruvate + NAD(P)H + CO_2_. A *Synechocystis* 6803 mutant which carries a transposon insertional mutation in the *me* gene exhibits poor growth under photoautotrophic conditions (23), and *me* gene-disrupted mutants cannot grow under dark heterotrophic conditions (15). These results suggest that ME is necessary for normal growth in cyanobacteria. However, biochemical characteristics of cyanobacterial ME, such as the malate oxidation activity, coenzyme specificity, and reaction specificity, have not yet been revealed. Thus, it is unclear whether ME oxidizes malate that originates from the TCA cycle in cyanobacteria.

In this study, we revealed that ME, not MDH, mainly oxidizes malate that originates from the TCA cycle in cyanobacteria using analyses focusing on ME and MDH.

## RESULTS

### *Sy*ME showed higher activity than *Sy*MDH for malate oxidation when using NADP^+^ as a coenzyme.

**FIG 1:**
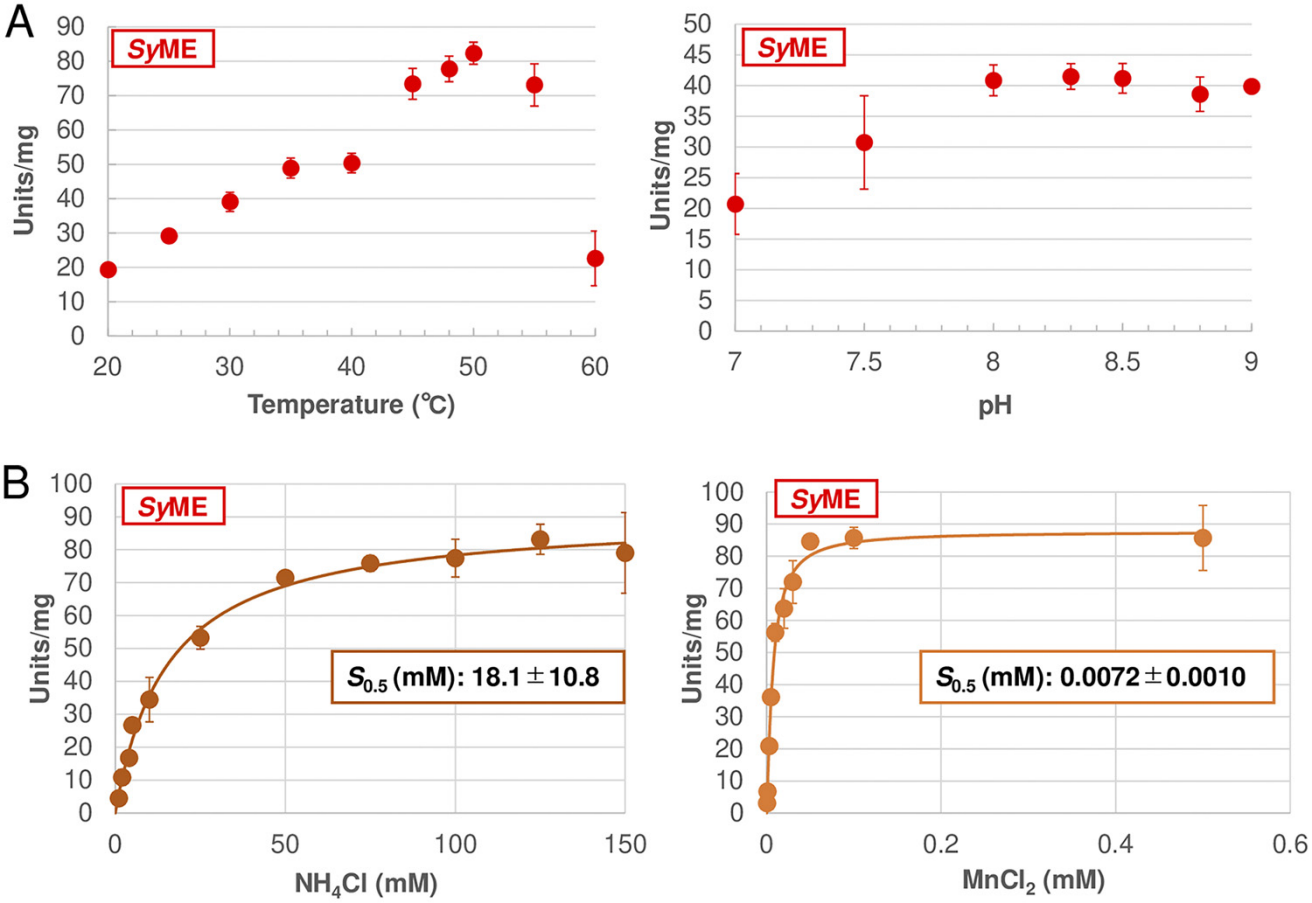
Optimization of measurement conditions of *Synechocystis* 6803 malic enzyme (ME) (*Sy*ME). (A) *Sy*ME activity at different temperatures (left) and pH values (right). In the measurement of the temperature dependence, the pH was fixed at 8.0. In the measurement of the pH dependence, the temperature was fixed at 30°C. The concentrations of malate, NADP^+^, NH_4_Cl, and MnCl_2_ were 2.0, 0.5, 50.0, and 1.0 mM, respectively. (B) *Sy*ME activity at different concentrations of NH_4_Cl (left) and MnCl_2_ (right). These measurements were performed at 50°C and pH 8.3. The concentrations of malate and NADP^+^ were 2.0 mM and 0.5 mM, respectively. In the measurement of the dependence of the NH_4_Cl concentration, the MnCl_2_ concentration was fixed at 1 mM. In the measurement of the dependence of the MnCl_2_ concentration, the NH_4_Cl concentration was fixed at 100 mM. Error analyses of the curve fittings revealed that the coefficients of determination (*R*^2^) of the saturation curves for NH_4_Cl and MnCl_2_ were 0.99391 and 0.99265, respectively. *S*_0.5_ indicates the half-saturation concentration of NH_4_Cl and MnCl_2_. All data plots are the mean ± standard deviation calculated from three independent experiments.

First, we performed biochemical analysis of *Sy*ME. *Sy*ME was purified as a His-tagged protein (see Fig. S1 in the supplemental material). *Sy*ME showed the highest activity at 50°C and pH 8.3 when NADP^+^ was utilized as a coenzyme (Fig. 1A). Similar to other bacterial MEs (24–26), *Sy*ME activity strongly depended on monovalent and divalent cations (particularly NH_4_^+^ and Mn^2+^) (Fig. S2). The *S*_0.5_ (half-saturation concentration) values of *Sy*ME for NH_4_Cl and MnCl_2_ were 18.1 mM and 0.0072 mM, respectively (Fig. 1B). Therefore, we defined the optimum conditions of *Sy*ME as follows: 50°C and pH 8.3 in the presence of 100 mM NH_4_Cl and 0.5 mM MnCl_2_.

10.1128/mbio.02187-22.1 FIG S1 Results of sodium dodecyl sulfate-polyacrylamide gel electrophoresis (SDS-PAGE) after purification of cyanobacterial enzymes. The purity of the protein was confirmed using 12% SDS-PAGE gels. Download FIG S1, TIF file, 0.2 MB. Copyright © 2022 Katayama et al. 2022 Katayama et al. https://creativecommons.org/licenses/by/4.0/ This content is distributed under the terms of the Creative Commons Attribution 4.0 International license.

10.1128/mbio.02187-22.2 FIG S2 Effect of monovalent and divalent cations on *Synechocystis* 6803 malic enzyme (ME) (*Sy*ME) activity. The measurement was performed at 30°C and pH 8.0. The concentrations of malate and NADP^+^ were 2.0 mM and 0.5 mM, respectively. The concentrations of monovalent and divalent cations were 50 mM and 1 mM, respectively. All cations used in this measurement were chloride. All data are the mean ± standard deviation calculated from three independent experiments. Download FIG S2, TIF file, 0.1 MB. Copyright © 2022 Katayama et al. 2022 Katayama et al. https://creativecommons.org/licenses/by/4.0/ This content is distributed under the terms of the Creative Commons Attribution 4.0 International license.

**FIG 2:**
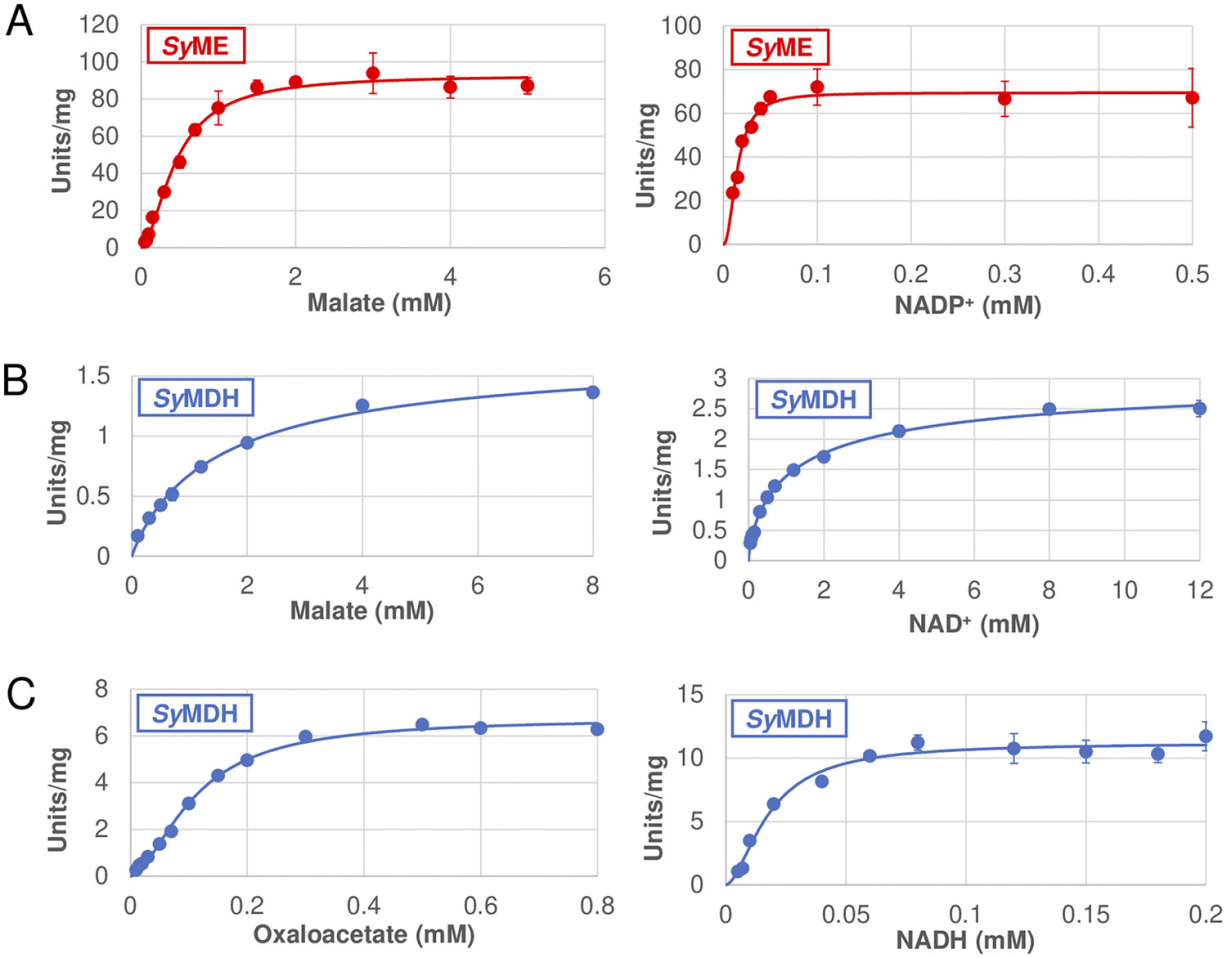
Saturation curves of *Synechocystis* 6803 malic enzyme (ME) (*Sy*ME) and *Synechocystis* 6803 malate dehydrogenase (MDH) (*Sy*MDH) for substrates and coenzymes. (A) Saturation curves of *Sy*ME for malate (left) and NADP^+^ (right). These measurements were performed at 50°C and pH 8.3. In the measurement of the saturation curve for malate, the NADP^+^ concentration was fixed at 0.5 mM. In the measurement of the saturation curve for NADP^+^, the malate concentration was fixed at 3 mM. The concentrations of NH_4_Cl and MnCl_2_ were 100 mM and 0.5 mM, respectively. Error analyses of the curve fittings revealed that the coefficients of determination (*R*^2^) of the saturation curves for malate and NADP^+^ were 0.99219 and 0.96929, respectively. (B) Saturation curves of *Sy*MDH for malate (left) and NAD^+^ (right). These measurements were performed under the optimum conditions of *Sy*MDH (50°C and pH 8.0) (18). In the measurement of the saturation curve for malate, the NAD^+^ concentration was fixed at 8 mM. In the measurement of the saturation curve for NAD^+^, the malate concentration was fixed at 4 mM. Error analyses of the curve fittings revealed that the *R*^2^ of the saturation curves for malate and NAD^+^ were 0.99445 and 0.99743, respectively. (C) Saturation curves of *Sy*MDH for oxaloacetate (left) and NADH (right). These measurements were performed at 50°C and pH 8.0. In the measurement of the saturation curve for oxaloacetate, the NADH concentration was fixed at 0.1 mM. In the measurement of the saturation curve for NADH, the oxaloacetate concentration was fixed at 0.5 mM. Error analyses of the curve fittings revealed that the *R*^2^ of the saturation curves for oxaloacetate and NADH were 0.99525 and 0.98133, respectively. All data plots are the mean ± standard deviation calculated from three independent experiments.

We compared the kinetic parameters under optimum conditions (*Sy*ME, 50°C and pH 8.3; *Sy*MDH, 50°C and pH 8.0) (18) between *Sy*ME and *Sy*MDH (Table 1). Similar to *Sy*ME, *Sy*MDH was purified as a His-tagged protein (Fig. S1). Both *Sy*ME and *Sy*MDH activities increased depending on the concentrations of malate and NAD(P)^+^ (Fig. 2A and B). The *S*_0.5_ and *k*_cat_ (turnover number) of *Sy*ME for malate were lower and higher than those of *Sy*MDH, respectively (Table 1). The *k*_cat_/*S*_0.5_ (catalytic efficiency) of *Sy*ME for malate was 264-fold higher than that of *Sy*MDH (Table 1). In contrast to *Sy*MDH, *Sy*ME specifically showed enzymatic activity for NADP^+^ rather than NAD^+^; the *k*_cat_/*S*_0.5_ for NADP^+^ was 437-fold higher than that for NAD^+^ (Table 1 and Table S2). The *k*_cat_/*S*_0.5_ of *Sy*ME for NADP^+^ was 2,673-fold higher than that of *Sy*MDH for NAD^+^ (Table 1). *Sy*ME also showed no enzymatic activity in the reductive reaction of the conversion of pyruvate to malate. However, *Sy*MDH showed high specificity for the reductive reaction of the conversion of oxaloacetate into malate (Fig. 2B and C); the *k*_cat_/*S*_0.5_ values for oxaloacetate and NADH were 57-fold and 264-fold higher than those for malate and NAD^+^, respectively (Table 1).

**TABLE 1:** Kinetic parameters of cyanobacterial malic enzymes and malate dehydrogenases under optimum conditions^*a*^

Reaction and enzyme	Substrate or coenzyme	*S*_0.5_ (mM)	*k*_cat_ (s^−1^)	*k*_cat_/*S*_0.5_ (s^−1^ mM^−1^)	*n*_H_
Malate oxidation					
*Sy*ME	Malate	0.46 ± 0.06*	77.2 ± 5.9**	169 ± 10**	1.76 ± 0.25*
	NADP^+^	0.015 ± 0.001*	55.7 ± 3.3**	3689 ± 23**	2.14 ± 0.29*
*Sy*MDH	Malate	1.61 ± 0.37	1.0 ± 0.1	0.64 ± 0.09	0.93 ± 0.06
	NAD^+^	1.34 ± 0.30	1.8 ± 0.1	1.4 ± 0.2	0.72 ± 0.03
*Ar*ME	Malate	0.30 ± 0.03*	42.8 ± 0.9**	144 ± 14**	1.15 ± 0.07*
	NADP^+^	0.034 ± 0.003**	49.5 ± 2.0**	1458 ± 69**	1.03 ± 0.11*
*Ar*MDH	Malate	0.034 ± 0.002	0.25 ± 0.004	7.3 ± 0.3	1.49 ± 0.08
	NAD^+^	0.30 ± 0.02	0.50 ± 0.01	1.7 ± 0.1	0.66 ± 0.01
*No*ME	Malate	0.41 ± 0.02*	30.3 ± 1.2**	75 ± 0.4**	1.57 ± 0.06**
	NADP^+^	0.014 ± 0.004*	33.4 ± 3.2**	2533 ± 436*	1.07 ± 0.28
*No*MDH	Malate	1.88 ± 0.56	3.5 ± 0.4	2.0 ± 0.4	0.86 ± 0.10
	NAD^+^	8.45 ± 1.95	6.3 ± 0.5	0.77 ± 0.10	0.75 ± 0.02

Reductive reaction					
*Sy*MDH	Oxaloacetate	0.11 ± 0.002*	4.0 ± 0.04**	36 ± 1**	1.71 ± 0.07**
	NADH	0.018 ± 0.002*	6.6 ± 0.5**	364 ± 19**	1.75 ± 0.27*
*Ar*MDH	Oxaloacetate	0.041 ± 0.001*	1.4 ± 0.03**	34 ± 0.4**	1.84 ± 0.08*
	NADH	0.0085 ± 0.0025**	1.7 ± 0.1**	214 ± 50*	1.14 ± 0.52
*No*ME	Pyruvate	6.55 ± 0.38**	0.79 ± 0.03**	0.12 ± 0.003**	1.52 ± 0.01
	NADPH	0.0074 ± 0.001	0.85 ± 0.03**	116 ± 10*	0.94 ± 0.09
*No*MDH	Oxaloacetate	0.026 ± 0.002*	1.1 ± 0.03*	42 ± 2**	1.65 ± 0.05**
	NADH	0.0093 ± 0.0001*	1.3 ± 0.1**	142 ± 8**	1.10 ± 0.05**

a The activities of *Sy*ME and *Ar*ME for the reductive reaction were not detected. These kinetic parameters represent the mean ± standard deviation calculated from three independent saturation curves. Asterisks above the kinetic parameters in malate oxidation represent statistically significant differences between the kinetic parameters of ME and MDH in malate oxidation obtained using Welch’s *t* test (*, *P* < 0.05; **, *P* < 0.005). Asterisks above the kinetic parameters in the reductive reaction represent statistically significant differences between the kinetic parameters in malate oxidation and the reductive reaction obtained from Welch’s *t* test (*, *P* < 0.05; **, *P* < 0.005). The explanation of each kinetic parameter is as follows: *S*_0.5_, half-saturation concentration (concentration at 50% *V*_max_); *k*_cat_, turnover number; *k*_cat_/*S*_0.5_, catalytic efficiency; *n*_H_, Hill coefficient.

10.1128/mbio.02187-22.9 TABLE S2 Kinetic parameters of cyanobacterial MEs for NAD^+^. The measurements of saturation curves for NAD^+^ were performed at the optimum conditions (*Sy*ME, 50°C and pH 8.3 in the presence of 100 mM NH_4_Cl and 0.5 mM MnCl_2_; *Ar*ME, 47°C and pH 8.5 in the presence of 100 mM NH_4_Cl and 0.1 mM MnCl_2_; *No*ME, 52°C and pH 8.8 in the presence of 100 mM NH_4_Cl and 0.1 mM MnCl_2_). The concentration of malate was fixed at 3 mM. These kinetic parameters represent the mean ± standard deviation calculated from three independent saturation curves. Asterisks represent statistically significant differences between the kinetic parameters of cyanobacterial MEs for NADP^+^ (Table 1) and NAD^+^ obtained from Welch’s *t* test (*, *P* < 0.05; **, *P* < 0.005). Download Table S2, DOCX file, 0.02 MB. Copyright © 2022 Katayama et al. 2022 Katayama et al. https://creativecommons.org/licenses/by/4.0/ This content is distributed under the terms of the Creative Commons Attribution 4.0 International license.

Similarly, the differences in the kinetic parameters between *Sy*ME and *Sy*MDH were confirmed at the optimum growth temperature of *Synechocystis* 6803 (30°C) (27) (Table 2). *Sy*ME showed higher catalytic efficiency in malate oxidation than *Sy*MDH when using NADP^+^ as a coenzyme (Table 2). In contrast to *Sy*ME, *Sy*MDH showed high specificity for the reductive reaction (Table 2).

**TABLE 2:** Kinetic parameters of *Synechocystis* 6803 malic enzyme and malate dehydrogenase at 30°C^*a*^

Reaction and enzyme	Substrate or coenzyme	*S*_0.5_ (mM)	*k*_cat_ (s^−1^)	*k*_cat_/*S*_0.5_ (s^−1^ mM^−1^)	*n*_H_
Malate oxidation					
*Sy*ME	Malate	0.92 ± 0.06**	39.1 ± 1.7**	43 ± 2**	2.74 ± 0.39*
	NADP^+^	0.014 ± 0.002*	42.1 ± 3.5**	3,017 ± 209**	1.17 ± 0.24
	NAD^+^	1.79 ± 0.27	2.46 ± 0.20	1.4 ± 0.1	1.69 ± 0.35
*Sy*MDH	Malate	0.23 ± 0.03	0.72 ± 0.02	3.1 ± 0.3	0.98 ± 0.04
	NAD^+^	0.35 ± 0.04	0.64 ± 0.02	1.8 ± 0.2	0.92 ± 0.06

Reductive reaction					
*Sy*MDH	Oxaloacetate	0.63 ± 0.07*	12.9 ± 0.5**	20 ± 1**	1.03 ± 0.02
	NADH	0.0071 ± 0.0019*	10.4 ± 1.1**	1,512 ± 274*	0.72 ± 0.17

a The measurements of saturation curves of *Sy*ME and *Sy*MDH were performed at the intracellular pH of *Synechocystis* 6803 (pH 7.8) (52). In the measurements of the saturation curves of *Sy*ME, the concentrations of NH_4_Cl and MnCl_2_ were fixed at 100 mM and 0.5 mM, respectively. In the measurement of the saturation curve of *Sy*ME for malate, the NADP^+^ concentration was fixed at 0.5 mM. In the measurement of the saturation curve of *Sy*ME for NAD(P)^+^, the malate concentration was fixed at 3 mM. The activity of *Sy*ME for the reductive reaction was not detected. In the measurement of the saturation curve of *Sy*MDH for malate, the NAD^+^ concentration was fixed at 8 mM. In the measurement of the saturation curve of *Sy*MDH for malate, the NAD^+^ concentration was fixed at 8 mM. In the measurement of the saturation curve of *Sy*MDH for NAD^+^, the malate concentration was fixed at 4 mM. In the measurement of the saturation curve of *Sy*MDH for oxaloacetate, the NADH concentration was fixed at 0.1 mM. In the measurement of the saturation curve of *Sy*MDH for NADH, the oxaloacetate concentration was fixed at 2 mM. These kinetic parameters represent the mean ± standard deviation calculated from three independent saturation curves. Asterisks above the kinetic parameters in malate oxidation represent statistically significant differences between the kinetic parameters of *Sy*ME and *Sy*MDH in malate oxidation obtained using Welch’s *t* test (*, *P* < 0.05; **, *P* < 0.005). Asterisks above the kinetic parameters in the reductive reaction represent statistically significant differences between the kinetic parameters in malate oxidation and the reductive reaction obtained from Welch’s *t* test (*, *P* < 0.05; **, *P* < 0.005).

### *Sy*ME, not *Sy*MDH, continuously catalyzed the sequential enzymatic reaction with fumarase.

**FIG 3:**
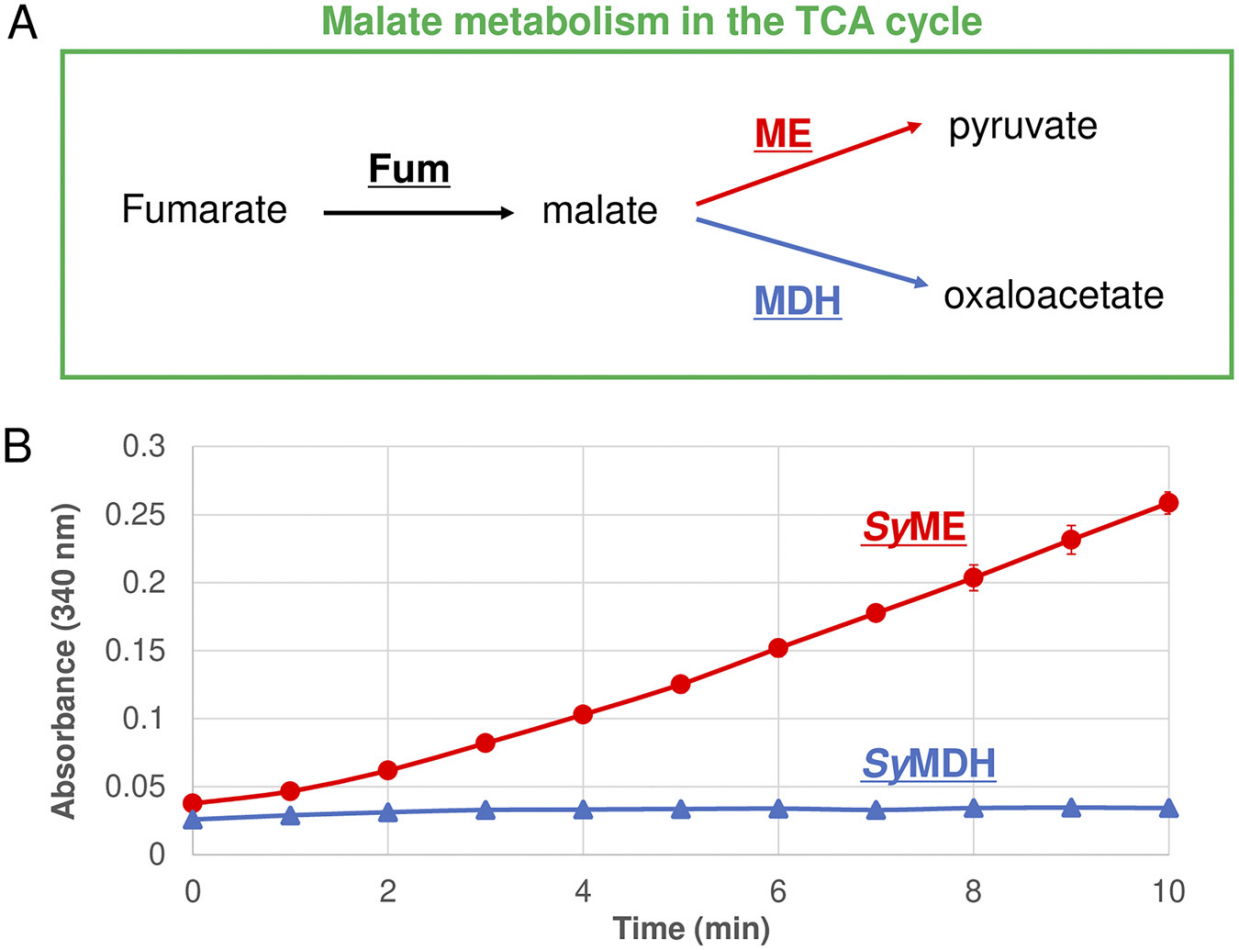
Coupled activity assays with fumarase (Fum) from *Synechocystis* 6803 (*Sy*Fum). (A) Model drawing of malate metabolism in the tricarboxylic acid (TCA) cycle. (B) Results of coupled activity assays with *Sy*Fum. The red circles and blue triangles indicate the results using *Synechocystis* 6803 malic enzyme (ME) (*Sy*ME) and *Synechocystis* 6803 malate dehydrogenase (MDH) (*Sy*MDH) as enzymes catalyzing malate oxidation, respectively. All data plots are the mean ± standard deviation calculated from three independent experiments.

In the TCA cycle of higher plants, the interaction between adjacent enzymes is often important for sequential enzymatic reactions (28). Malate is generated from fumarate via a reaction catalyzed by fumarase (Fum) in the TCA cycle (Fig. 3A). We performed a coupled activity assay using Fum from *Synechocystis* 6803 (*Sy*Fum) at room temperature (23°C) (Fig. 3B). *Sy*Fum was purified as a His-tagged protein (Fig. S1). After adding fumarate to the reaction mixture as a starting substrate, we monitored the amount of NAD(P)H produced by measuring the absorbance at 340 nm (Fig. 3B). When *Sy*ME was used as a malate oxidation enzyme, the absorbance at 340 nm increased over time; that is, the sequential enzymatic reaction with *Sy*Fum proceeded continuously (Fig. 3B). However, when *Sy*MDH was used, the absorbance at 340 nm hardly changed; that is, the sequential enzymatic reaction with *Sy*Fum did not proceed (Fig. 3B).

### *Synechocystis* 6803 strain lacking the *me* gene (ΔME strain) accumulated malate in the cells.

**FIG 4:**
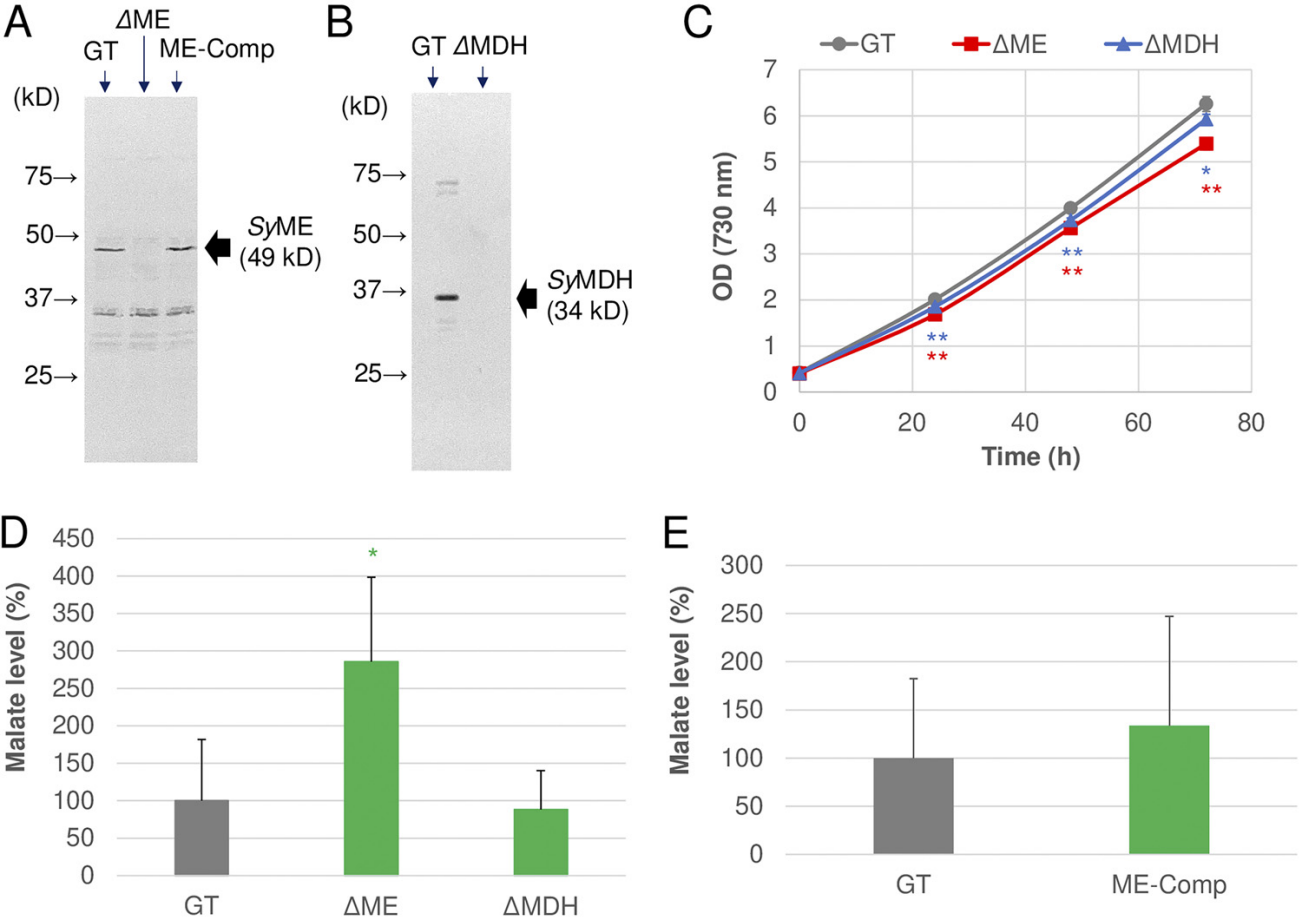
Intracellular malate levels in *Synechocystis* 6803 mutants. (A) Protein levels of *Synechocystis* 6803 malic enzyme (ME) (*Sy*ME) in glucose-tolerant (GT) strain, *Synechocystis* 6803 strain lacking the *me* gene (ΔME), and ME-complement strain (ME-Comp). Proteins after aerobic cultivation were subjected to immunoblotting. In sodium dodecyl sulfate-polyacrylamide gel electrophoresis (SDS-PAGE), 20 μg of proteins was used. (B) Protein levels of *Synechocystis* 6803 malate dehydrogenase (MDH) (*Sy*MDH) in GT strain and *Synechocystis* 6803 strain lacking the *citH* gene (ΔMDH). Proteins after photoautotrophic cultivation were subjected to immunoblotting. In SDS-PAGE, 6 μg of proteins was used. (C) Growth curves of GT, ΔME, and ΔMDH strains under aerobic conditions. (D) Intracellular malate levels in GT, ΔME, and ΔMDH strains after photoautotrophic cultivation. The malate levels are represented by a relative value, and that in the GT strain was set at 100%. (E) Intracellular malate levels in GT and ME-Comp strains after photoautotrophic cultivation. The malate levels are represented by a relative value, and that in the GT strain was set at 100%. All data are the mean ± standard deviation calculated from three or four independent experiments. All asterisks represent statistically significant differences between GT and the mutant strains obtained from Welch’s *t* test (*, *P* < 0.05; **, *P* < 0.005).

To compare the catalytic activities of *Sy*ME and *Sy*MDH *in vivo*, we constructed a *Synechocystis* 6803 strain lacking the *me* gene (ΔME strain) and *citH* gene encoding *Sy*MDH (ΔMDH strain) (Fig. 4A and B) and measured the intracellular malate levels in these mutants. Since the deletion of the *me* gene in *Synechocystis* 6803 makes it impossible to grow under dark heterotrophic conditions (15), these mutants were cultivated under photoautotrophic conditions (Fig. 4C). The intracellular malate level in the ΔME strain was approximately 3-fold higher than that in the wild-type glucose-tolerant (GT) strain of *Synechocystis* 6803 (Fig. 4D). In contrast, the intracellular malate level in the ΔMDH strain was approximately the same as that in the GT strain (Fig. 4D). To confirm the malate accumulation in the ΔME strain, we introduced the *me* gene into the ΔME strain, i.e., the ME-complement (ME-Comp) strain (Fig. 4A). The intracellular malate level in the ME-Comp strain was the same as that in the GT strain (Fig. 4E).

### ME was highly conserved in cyanobacterial genomes compared with MDH.

**FIG 5:**
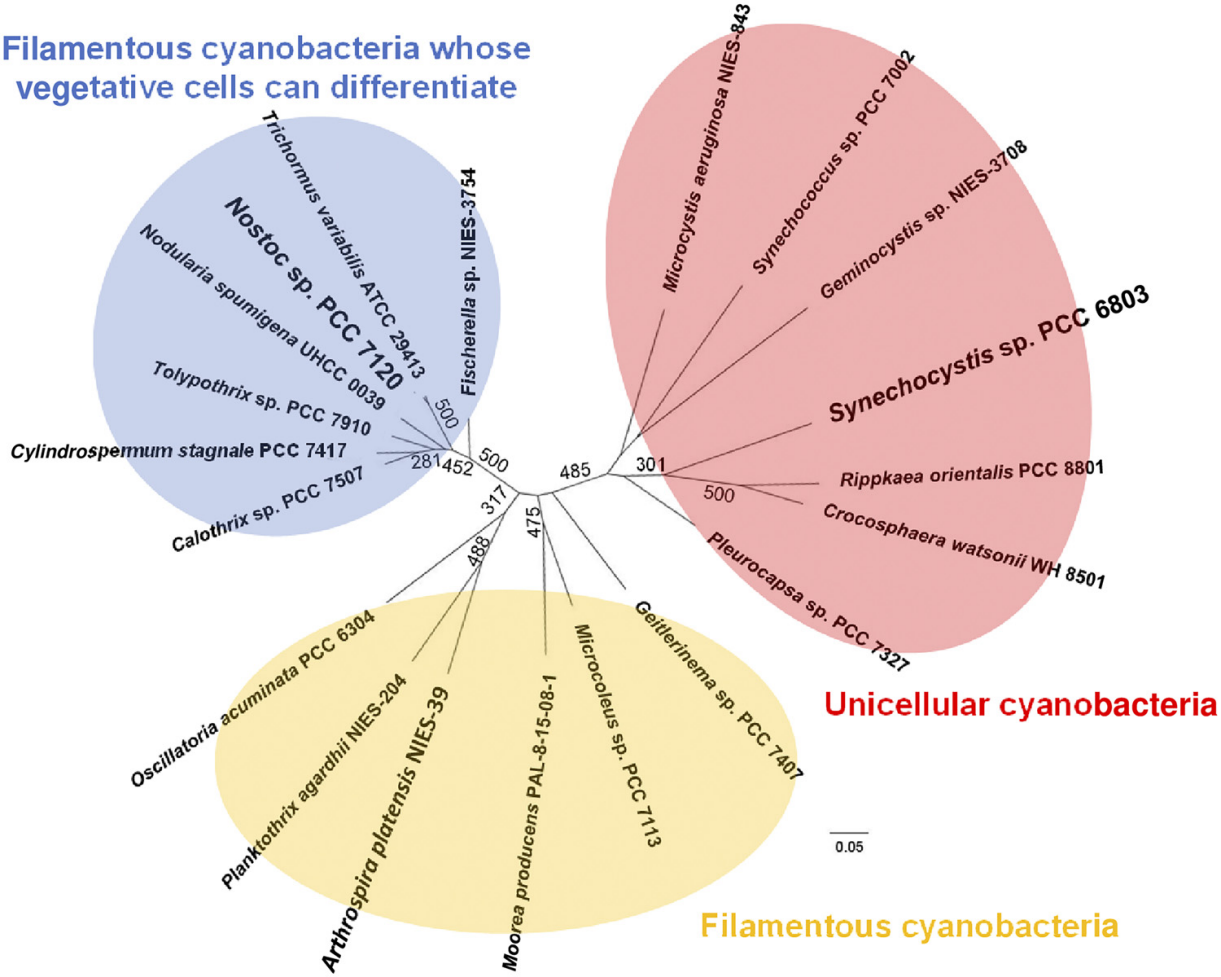
Phylogenetic tree of malic enzymes (MEs) in cyanobacteria that possess both ME and malate dehydrogenase (MDH). Multiple sequence alignment of cyanobacterial MEs that include 463 conserved amino acids was performed by CLC Sequence Viewer ver. 8.0. Based on the alignment results, the maximum-likelihood tree was generated at PHYML online (http://www.atgc-montpellier.fr/phyml/). The bootstrap values were obtained from 500 replications, and values over 250 are displayed in the figure. Accession numbers (NCBI protein identifier [ID]) of these enzymes are listed in Table S3.

Our biochemical analysis indicated that ME had higher malate oxidation activity than MDH in *Synechocystis* 6803. We performed bioinformatic analyses (BLAST analysis and phylogenetic analysis) using cyanobacterial ME and MDH sequences to examine the conservation of these enzymes among cyanobacteria (Table S3; Fig. 5 and Fig. S3). BLAST analyses of ME and MDH for all sequenced cyanobacteria (130 species) revealed that 78% (102 species) and 51% (66 species) of the sequenced cyanobacteria possessed ME and MDH, respectively (Table S3). In addition, 51% (66 species) possessed both ME and MDH; that is, none of the cyanobacteria possessed only MDH (Table S3). Cyanobacteria are morphologically divided into unicellular and filamentous types. In some filamentous cyanobacteria, vegetative cells can differentiate into specialized cells to survive environmental stress (29). Phylogenetic analysis of cyanobacterial MEs revealed that the amino acid sequences of cyanobacterial MEs were highly preserved per each group based on the cyanobacterial morphological characteristics (Fig. 5). The amino acid sequences of cyanobacterial MDHs were also highly preserved per each group (Fig. S3).

10.1128/mbio.02187-22.3 FIG S3 Phylogenetic tree of malate dehydrogenases (MDHs) in cyanobacteria. Multiple sequence alignment of cyanobacterial MDHs that include 279 conserved amino acids was performed by CLC Sequence Viewer ver. 8.0. Based on the alignment results, the maximum-likelihood tree was generated at PHYML online (http://www.atgc-montpellier.fr/phyml/). The bootstrap values were obtained from 500 replications, and values over 250 are displayed in the figure. Accession numbers (NCBI protein ID) of these enzymes are listed in Table S2. Download FIG S3, TIF file, 0.2 MB. Copyright © 2022 Katayama et al. 2022 Katayama et al. https://creativecommons.org/licenses/by/4.0/ This content is distributed under the terms of the Creative Commons Attribution 4.0 International license.

10.1128/mbio.02187-22.10 TABLE S3 Results of BLAST analyses of ME and MDH for the sequenced cyanobacteria. A plus sign indicates that each cyanobacterium possesses ME or MDH. BLAST searches of ME and MDH for all sequenced cyanobacteria (130 species) registered in the KEGG genes database (https://www.genome.jp/kegg/genes.html) were conducted using BLASTP in January 2021. The amino acid sequences of *Sy*ME (accession number BAA16663) and *Sy*MDH (accession number BAA10470) were used as query sequences in the BLAST search of ME and MDH, respectively. The threshold in the BLAST search of both ME and MDH was set at 10^-10^. The sequences of MDH and l-lactate dehydrogenase having high homology to MDH were distinguished by five amino acid residues that determine the substrate specificity of these enzymes (Y. Yin and J. F. Kirsch, Proc Natl Acad Sci U S A 104:17353–17357, 2007, https://doi.org/10.1073/pnas.0708265104). Download Table S3, XLSX file, 0.02 MB. Copyright © 2022 Katayama et al. 2022 Katayama et al. https://creativecommons.org/licenses/by/4.0/ This content is distributed under the terms of the Creative Commons Attribution 4.0 International license.

### Differences in biochemical characteristics between ME and MDH observed in *Synechocystis* 6803 were conserved in other cyanobacteria.

Finally, we performed biochemical analyses of MEs and MDHs from two cyanobacterial species, namely, Arthrospira platensis NIES-39 and *Nostoc* sp. strain PCC 7120, which belong to the other groups based on the cyanobacterial morphological characteristics (Fig. 5 and Fig. S3). They were purified as glutathione *S*-transferase (GST)-tagged proteins (Fig. S1). Biochemical analyses of these enzymes were generally conducted under each optimum condition; however, the measurement temperature of MDHs was standardized to the optimum temperature of *Sy*MDH (50°C) to avoid the decomposition of oxaloacetate under high-temperature conditions (Fig. S4 and S5).

10.1128/mbio.02187-22.4 FIG S4 Optimization of measurement conditions of *Arthrospira platensis* NIES-39 malic enzyme (*Ar*ME) and *Arthrospira platensis* NIES-39 malate dehydrogenase (*Ar*MDH). (A) *Ar*ME activity at different temperatures (left) and pH values (right). In the measurement of the temperature dependence, the pH was fixed at 8.0. In the measurement of the pH dependence, the temperature was fixed at 30°C. The concentrations of malate, NADP^+^, NH_4_Cl, and MnCl_2_ were 2.0, 0.5, 50.0, and 1.0 mM, respectively. (B) *Ar*ME activity at different concentrations of NH_4_Cl (left) and MnCl_2_ (right). These measurements were performed at 47°C and pH 8.5. The concentrations of malate and NADP^+^ were 2.0 mM and 0.5 mM, respectively. In the measurement of the dependence of the NH_4_Cl concentration, the MnCl_2_ concentration was fixed at 1 mM. In the measurement of the dependence of the MnCl_2_ concentration, the NH_4_Cl concentration was fixed at 100 mM. Error analyses of the curve fittings revealed that the coefficients of determination (*R*^2^) of the saturation curves for NH_4_Cl and MnCl_2_ were 0.95528 and 0.99509, respectively. (C) *Ar*MDH activity at different temperatures (left) and pH values (right). In the measurement of the temperature dependence, the pH was fixed at 8.0. In the measurement of the pH dependence, the temperature was fixed at 30°C. The concentrations of malate and NAD^+^ were 4 mM and 8 mM, respectively. All data plots are the mean ± standard deviation calculated from three independent experiments. Download FIG S4, TIF file, 0.1 MB. Copyright © 2022 Katayama et al. 2022 Katayama et al. https://creativecommons.org/licenses/by/4.0/ This content is distributed under the terms of the Creative Commons Attribution 4.0 International license.

10.1128/mbio.02187-22.5 FIG S5 Optimization of measurement conditions of *Nostoc* sp. PCC 7120 malic enzyme (*No*ME) and *Nostoc* sp. PCC 7120 malate dehydrogenase (*No*MDH). (A) *No*ME activity at different temperatures (left) and pH values (right). In the measurement of the temperature dependence, the pH was fixed at 8.0. In the measurement of the pH dependence, the temperature was fixed at 30°C. The concentrations of malate, NADP^+^, NH_4_Cl, and MnCl_2_ were 2.0, 0.5, 50.0, and 1.0 mM, respectively. (B) *No*ME activity at different concentrations of NH_4_Cl (left) and MnCl_2_ (right). These measurements were performed at 52°C and pH 8.8. The concentrations of malate and NADP^+^ were 2.0 mM and 0.5 mM, respectively. In the measurement of the dependence of the NH_4_Cl concentration, the MnCl_2_ concentration was fixed at 1 mM. In the measurement of the dependence of the MnCl_2_ concentration, the NH_4_Cl concentration was fixed at 100 mM. Error analyses of the curve fittings revealed that the coefficients of determination (*R*^2^) of the saturation curves for NH_4_Cl and MnCl_2_ were 0.99161 and 0.99525, respectively. (C) *No*MDH activity at different temperatures (left) and pH values (right). In the measurement of the temperature dependence, the pH was fixed at 8.0. In the measurement of the pH dependence, the temperature was fixed at 30°C. The concentrations of malate and NAD^+^ were 4 mM and 8 mM, respectively. All data plots are the mean ± standard deviation calculated from three independent experiments. Download FIG S5, TIF file, 0.1 MB. Copyright © 2022 Katayama et al. 2022 Katayama et al. https://creativecommons.org/licenses/by/4.0/ This content is distributed under the terms of the Creative Commons Attribution 4.0 International license.

The kinetic parameters of *Arthrospira platensis* NIES-39 and *Nostoc* sp. PCC 7120 enzymes (Table 1) were calculated based on their saturation curves (Fig. S6 and S7). The *k*_cat_/*S*_0.5_ of *Arthrospira platensis* NIES-39 ME (*Ar*ME) for malate was 20-fold higher than that of *Arthrospira platensis* NIES-39 MDH (*Ar*MDH) (Table 1). *Ar*ME and *Ar*MDH specifically used NADP^+^ and NAD^+^, respectively, as coenzymes (Table 1 and Table S2). The *k*_cat_/*S*_0.5_ of *Ar*ME for NADP^+^ was 858-fold higher than that of *Ar*MDH for NAD^+^ (Table 1). *Ar*ME showed no enzymatic activity for the reductive reaction (Table 1). In contrast, *Ar*MDH showed high specificity for the reductive reaction (Table 1). The *k*_cat_/*S*_0.5_ of *Nostoc* sp. PCC 7120 ME (*No*ME) for malate was 38-fold higher than that of *Nostoc* sp. PCC 7120 MDH (*No*MDH) (Table 1). *No*ME and *No*MDH specifically used NADP^+^ and NAD^+^, respectively, as coenzymes (Table 1 and Table S2). The *k*_cat_/*S*_0.5_ of *No*ME for NADP^+^ was 3,290-fold higher than that of *No*MDH for NAD^+^ (Table 1). *No*ME and *No*MDH showed a high specificity for oxidative and reductive reactions, respectively (Table 1).

10.1128/mbio.02187-22.6 FIG S6 Saturation curves of *Arthrospira platensis* NIES-39 malic enzyme (*Ar*ME) and *Arthrospira platensis* NIES-39 malate dehydrogenase (*Ar*MDH) for substrates and coenzymes. (A) Saturation curves of *Ar*ME for malate (left) and NADP^+^ (right). These measurements were performed at 47°C and pH 8.5. In the measurement of the saturation curve for malate, the NADP^+^ concentration was fixed at 0.5 mM. In the measurement of the saturation curve for NADP^+^, the malate concentration was fixed at 3 mM. The concentrations of NH_4_Cl and MnCl_2_ were 100 mM and 0.1 mM, respectively. Error analyses of the curve fittings revealed that the coefficients of determination (*R*^2^) of the saturation curves for malate and NADP^+^ were 0.99472 and 0.99539, respectively. (B) Saturation curves of *Ar*MDH for malate (left) and NAD^+^ (right). These measurements were performed at 50°C and pH 8.5. In the measurement of the saturation curve for malate, the NAD^+^ concentration was fixed at 8 mM. In the measurement of the saturation curve for NAD^+^, the malate concentration was fixed at 1 mM. Error analyses of the curve fittings revealed that the *R*^2^ of the saturation curves for malate and NAD^+^ were 0.96492 and 0.97044, respectively. (C) Saturation curves of *Ar*MDH for oxaloacetate (left) and NADH (right). These measurements were performed at 50°C and pH 8.5. In the measurement of the saturation curve for oxaloacetate, the NADH concentration was fixed at 0.1 mM. In the measurement of the saturation curve for NADH, the oxaloacetate concentration was fixed at 0.5 mM. Error analyses of the curve fittings revealed that the *R*^2^ of the saturation curves for oxaloacetate and NADH were 0.95744 and 0.9913, respectively. All data plots are the mean ± standard deviation calculated from three independent experiments. Download FIG S6, TIF file, 0.1 MB. Copyright © 2022 Katayama et al. 2022 Katayama et al. https://creativecommons.org/licenses/by/4.0/ This content is distributed under the terms of the Creative Commons Attribution 4.0 International license.

10.1128/mbio.02187-22.7 FIG S7 Saturation curves of *Nostoc* sp. PCC 7120 malic enzyme (*No*ME) and *Nostoc* sp. PCC 7120 malate dehydrogenase (*No*MDH) for substrates and coenzymes. (A) Saturation curves of *No*ME for malate (left) and NADP^+^ (right). These measurements were performed at 52°C and pH 8.8. In the measurement of the saturation curve for malate, the NADP^+^ concentration was fixed at 0.5 mM. In the measurement of the saturation curve for NADP^+^, the malate concentration was fixed at 3 mM. The concentrations of NH_4_Cl and MnCl_2_ were 100 mM and 0.1 mM, respectively. Error analyses of the curve fittings revealed that the coefficients of determination (*R*^2^) of the saturation curves for malate and NADP^+^ were 0.99768 and 0.99026, respectively. (B) Saturation curves of *No*ME for pyruvate (left) and NADPH (right). These measurements were performed at 52°C and pH 8.8. In the measurement of the saturation curve for pyruvate, the NADPH concentration was fixed at 0.1 mM. In the measurement of the saturation curve for NADPH, the pyruvate concentration was fixed at 30 mM. The concentrations of NH_4_Cl, MnCl_2_, and KHCO_3_ were 100, 0.1, and 50 mM, respectively. Error analyses of the curve fittings revealed that the *R*^2^ of the saturation curves for pyruvate and NADPH were 0.96841 and 0.97779, respectively. (C) Saturation curves of *No*MDH for malate (left) and NAD^+^ (right). These measurements were performed at 50°C and pH 9.0. In the measurement of the saturation curve for malate, the NAD^+^ concentration was fixed at 8 mM. In the measurement of the saturation curve for NAD^+^, the malate concentration was fixed at 5 mM. Error analyses of the curve fittings revealed that the *R*^2^ of the saturation curves for malate and NAD^+^ were 0.99301 and 0.98286, respectively. (D) Saturation curves of *No*MDH for oxaloacetate (left) and NADH (right). These measurements were performed at 50°C and pH 9.0. In the measurement of the saturation curve for oxaloacetate, the NADH concentration was fixed at 0.1 mM. In the measurement of the saturation curve for NADH, the oxaloacetate concentration was fixed at 0.1 mM. Error analyses of the curve fittings revealed that the *R*^2^ of the saturation curves for oxaloacetate and NADH were 0.9331 and 0.98853, respectively. All data plots are the mean ± standard deviation calculated from three independent experiments. Download FIG S7, TIF file, 0.1 MB. Copyright © 2022 Katayama et al. 2022 Katayama et al. https://creativecommons.org/licenses/by/4.0/ This content is distributed under the terms of the Creative Commons Attribution 4.0 International license.

## DISCUSSION

In this study, we examined which enzyme oxidizes malate that originates from the TCA cycle in cyanobacteria.

*Sy*ME showed markedly higher catalytic efficiency in malate oxidation than *Sy*MDH at both 50°C and 30°C (Tables 1 and 2). The absolute intracellular concentration (molar concentration) of malate has not been reported in cyanobacteria but has been reported in Escherichia coli (1.7 mM) (30). Although *Sy*ME showed a lower affinity for malate than *Sy*MDH at 30°C, the *S*_0.5_ of both enzymes (*Sy*ME, 0.92 mM; *Sy*MDH, 0.23 mM) was lower than the intracellular concentration of malate in E. coli (Table 2). These results mean that the difference in the affinity for malate between *Sy*ME and *Sy*MDH hardly affects the difference in the malate oxidation activity between *Sy*ME and *Sy*MDH *in vivo*. Therefore, we consider that *Sy*ME shows higher malate oxidation activity than *Sy*MDH *in vivo*. Similar to isocitrate dehydrogenase and succinic semialdehyde dehydrogenase from *Synechocystis* 6803 (16, 21), *Sy*ME showed high specificity for NADP^+^ rather than NAD^+^ (Tables 1 and 2 and see Table S2 in the supplemental material). In *Synechocystis* 6803, the intracellular concentration of NADP^+^ (0.614 μmol/g dry cell weight) is approximately the same as that of NAD^+^ (0.514 μmol/g dry cell weight) (31). These results indicate that *Sy*ME uses NADP^+^, not NAD^+^, as a coenzyme *in vivo*. Unlike MEs from higher plants (32–34), *Sy*ME showed no enzymatic activity for the reductive reaction (Tables 1 and 2). In higher plants, the TCA cycle enzymes form protein complexes (metabolons), and channeling of metabolites is performed between adjacent enzymes (28). The results of the coupled activity assay with *Sy*Fum suggested that channeling of malate was not performed between *Sy*MDH and *Sy*Fum (Fig. 3B). Thus, *Sy*ME has more suitable properties for oxidizing malate that originates from the TCA cycle than *Sy*MDH.

**FIG 6:**
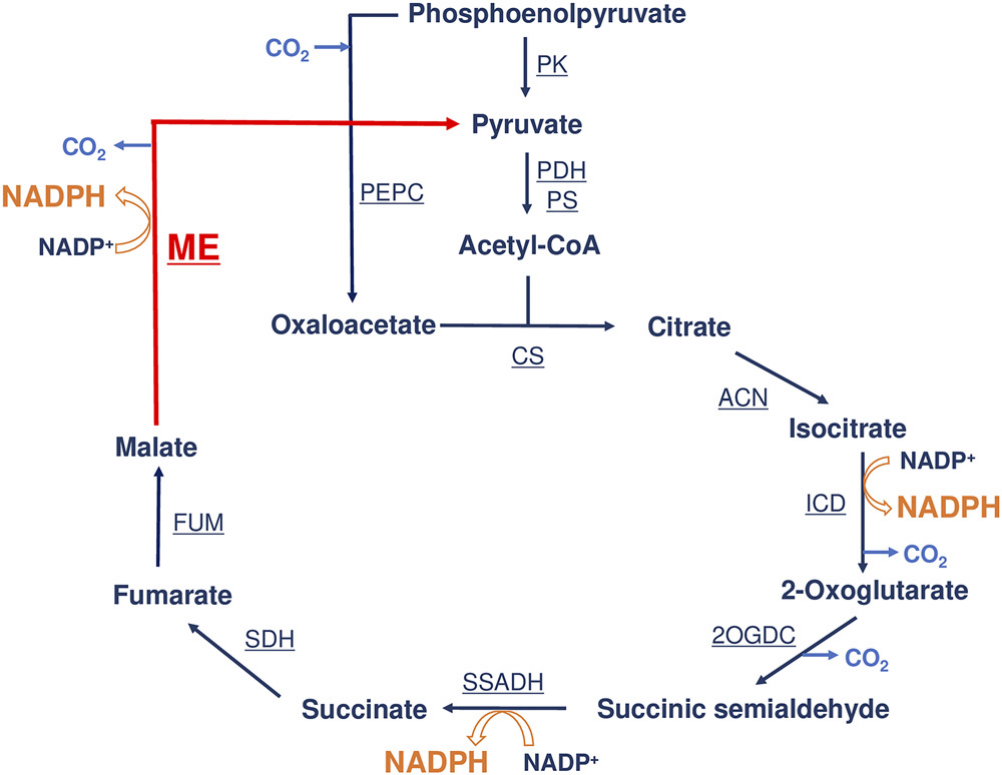
Schematic model of the malic enzyme (ME)-dependent tricarboxylic acid (TCA) cycle in cyanobacteria. PEPC, phosphoenolpyruvate carboxylase; PK, pyruvate kinase; PDH, pyruvate dehydrogenase; PS, pyruvate synthase; CS, citrate synthase; ACN, aconitase; ICD, isocitrate dehydrogenase; 2OGDC, 2-oxoglutarate decarboxylase; SSADH, succinic semialdehyde dehydrogenase; SDH, succinate dehydrogenase; FUM, fumarase.

Unlike the deletion of the *citH* gene, deletion of the *me* gene increased the intracellular malate level under photoautotrophic conditions (Fig. 4D). Previous metabolic flux analysis in *Synechocystis* 6803 revealed that under photoautotrophic conditions, there are fluxes from malate both to oxaloacetate and to pyruvate (35). *Synechocystis* 6803 accumulates malate in the cells under photoautotrophic conditions rather than dark conditions (36). These results suggest that under photoautotrophic conditions, *Sy*ME constantly catalyzes malate oxidation, while *Sy*MDH can catalyze malate oxidation against its reaction specificity when malate is accumulated in the cells. In addition, previous metabolic flux analyses in *Synechocystis* 6803 revealed that under mixotrophic conditions (37) and nitrogen-limited conditions (35), the reaction between malate and oxaloacetate and that between malate and pyruvate proceed in reductive and oxidative directions, respectively. These results reflect the differences in biochemical characteristics between *Sy*ME and *Sy*MDH revealed in this study. From the above, we concluded that *Sy*ME, but not *Sy*MDH, mainly oxidizes malate that originates from the TCA cycle (named the ME-dependent TCA cycle) (Fig. 6). In the ME-dependent TCA cycle, oxaloacetate is generated from phosphoenolpyruvate via the reaction catalyzed by phosphoenolpyruvate carboxylase (PEPC), which all photoautotrophs possess (Fig. 6).

The results of the BLAST analyses of ME and MDH in cyanobacteria suggested that ME is a more important enzyme for cyanobacterial carbon metabolism than MDH (Table S3). Cyanobacteria that possess only ME, not MDH, are candidates for exclusively possessing the ME-dependent TCA cycle, not the classical TCA cycle (named the MDH-dependent TCA cycle). However, to prove that only the ME-dependent TCA cycle acts in these cyanobacteria, further *in vivo* analyses such as metabolic flux analysis are also required. In the cyanobacteria that possess both ME and MDH, the differences in the biochemical characteristics of ME and MDH, such as the malate oxidation activity, coenzyme specificity, and reaction specificity, were highly preserved (Table 1). These results suggest that the ME-dependent TCA cycle acts as an alternative to the MDH-dependent TCA cycle in these cyanobacteria.

The ME-dependent TCA cycle generates three molecules of NADPH rather than NADH (Fig. 6). In cyanobacteria, both the respiratory chain and the photosynthetic electron transport chain are present on the thylakoid membrane (38, 39). Cyanobacterial NAD(P)H dehydrogenase (complex І), which is involved not only in oxidative phosphorylation but also in cyclic electron flow during photosynthesis, receives electrons from the reduced ferredoxin (40). Cyanobacterial ferredoxin-NADP^+^ reductase uses NADPH, not NADH, as a coenzyme (41, 42). Thus, NADPH is utilized as an electron carrier in complex І through ferredoxin reduction in the cyanobacterial respiratory chain. This is consistent with our opinion that the cyanobacterial TCA cycle is an NADPH-generating pathway. In cyanobacteria, the oxidative pentose phosphate (OPP) pathway also acts as an NADPH-generating pathway under dark conditions (15, 43). In *Synechocystis* 6803, the OPP pathway metabolites accumulate immediately after a shift from light to dark conditions (44). In contrast, in the *Synechocystis* 6803 mutant lacking the enzyme that catalyzes the first reaction in the OPP pathway, TCA cycle metabolites accumulate immediately after a shift from light to dark conditions (44). The OPP pathway enzymes catalyzing NADPH-generating reactions in *Synechocystis* 6803 are strongly inhibited by citrate in the TCA cycle (21, 45). Therefore, we consider that when the flux through the OPP pathway decreases owing to the accumulation of citrate, the flux through the ME-dependent TCA cycle increases to generate NADPH.

In the ME-dependent TCA cycle, malate is converted to pyruvate (Fig. 6). In cyanobacteria, pyruvate kinase (PK), which catalyzes the reaction that produces pyruvate from phosphoenolpyruvate, is inhibited by ATP, which is abundantly generated via the light reaction of photosynthesis (46, 47). Under photoautotrophic conditions, the *Synechocystis* 6803 mutant, which carries a transposon insertional mutation in the *me* gene, exhibits a lower growth rate than the GT strain; however, the growth rate in the mutant is restored by the addition of pyruvate (23). These results indicate that the ME-catalyzed reaction is important for pyruvate generation under photoautotrophic conditions in cyanobacteria. Under photoautotrophic conditions, not only the TCA cycle but also purine and urea metabolism generates fumarate in *Synechocystis* 6803 (12). However, previous metabolic flux analysis in *Synechocystis* 6803 revealed that under photoautotrophic conditions, the flux from succinate to fumarate in the TCA cycle is 4-fold higher than those from the other metabolites to fumarate (35). Therefore, we presume that under photoautotrophic conditions, the ME-dependent TCA cycle acts independently without being significantly affected by purine and urea metabolism.

In this study, we revealed that ME, but not MDH, mainly oxidizes malate that originates from the cyanobacterial TCA cycle. This finding suggests that the enzymes involved in the TCA cycle vary from organism to organism, and analyses focusing on enzymes, such as those performed in this study, are necessary to identify the reactions related to the TCA cycle.

## MATERIALS AND METHODS

### Construction of expression vectors that were transformed into E. coli cells.

The genomic regions containing genes encoding *Sy*ME (slr0721), *Sy*MDH (sll0891), *Sy*Fum (slr0018), *Ar*ME (NIES39_A01760), *Ar*MDH (NIES39_M01090), *No*ME (alr4596), and *No*MDH (alr4322) were commercially synthesized by Eurofin Genomics Japan (Tokyo, Japan). The regions containing the genes encoding *Sy*ME, *Sy*MDH, and *Sy*FUM were cloned into the BamHI-XhoI site of the pQE80L vector (Qiagen, Venlo, Netherlands). The regions containing the genes encoding *Ar*ME, *Ar*MDH, *No*ME, and *No*MDH were cloned into the BamHI-XhoI site of the pGEX6P-1 vector (GE Healthcare, Little Chalfont, UK).

### Affinity purification of His-tagged proteins.

The expression vectors containing the His-tagged *Synechocystis* 6803 enzyme (*Sy*ME, *Sy*MDH, or *Sy*Fum) were transformed into E. coli BL21(DE3) competent cells (BioDynamics Laboratory Inc., Tokyo, Japan). Thereafter, a shaking culture of 3.2 L of E. coli cells was conducted in LB medium overnight (25°C and 150 rpm) while inducing the expression of the His-tagged protein with 1 μM isopropyl-β-d-1-thiogalactopyranoside (Wako Chemicals, Osaka, Japan). After shaking culture, the E. coli cells were transferred to 20 mL of equilibrating buffer (20 mM Tris-HCl and 500 mM NaCl; pH 8.0) and disrupted by sonication (model VC-750; Eyela, Tokyo, Japan) for 2.5 min at 20% intensity. The suspension was then centrifuged (9,100 × *g* for 20 min at 4°C), and 1.5 mL of Talon metal affinity resin (TaKaRa Bio, Shiga, Japan) was added to the soluble fraction containing the His-tagged protein. The mixture was shaken gently for 30 min on ice to absorb the His-tagged protein in the resin. To remove the supernatant, the mixture was centrifuged (2,300 × *g* for 5 min at 4°C), and the residual resin bound to the His-tagged protein was washed five times using 1 mL of washing buffer (20 mM Tris-HCl, 500 mM NaCl, and 5 mM imidazole; pH 8.0). Subsequently, the resin was washed five times using 1 mL of equilibrating buffer. After the His-tagged protein was eluted five times in 700 μL of His tag elution buffer (20 mM Tris-HCl [pH 8.0], 500 mM NaCl, and 150 mM imidazole), it was concentrated using a Vivaspin 500 30-kDa-molecular-weight-cutoff (MWCO) concentrator (GE Healthcare, Chicago, IL, USA).

### Affinity purification of GST-tagged proteins.

The expression vectors containing each GST-tagged cyanobacterial enzyme (*Ar*ME, *Ar*MDH, *No*ME, or *No*MDH) were transformed into E. coli BL21(DE3) competent cells. In the purification of *Ar*ME, *Ar*MDH, and *No*ME, a shaking culture of 3.2 L of E. coli cells was conducted in LB medium overnight (25°C and 150 rpm) while inducing the expression of the GST-tagged protein with 1 μM isopropyl-β-d-1-thiogalactopyranoside. In the purification of *No*MDH, a shaking culture of 3.2 L of E. coli cells was performed in LB medium for 5 days (10°C and 150 rpm) while inducing the expression with 1 μM isopropyl-β-d-1-thiogalactopyranoside. The shaken E. coli cells were transferred to 20 mL of PBS-T (0.137 M NaCl, 2.7 mM KCl, 8.1 mM Na_2_HPO_4_·12H_2_O, 1.47 mM KH_2_PO_4_, 0.005% Tween 20). E. coli cells in PBS-T were disrupted by sonication (model VC-750) for 3.5 min at 20% intensity. The suspension was then centrifuged (5,800 × *g* for 10 min at 4°C), and 1 mL of glutathione-Sepharose 4B resin (GE Healthcare Japan, Tokyo, Japan) was added to the soluble fraction, including the GST-tagged protein. The mixture was shaken gently for 30 min on ice to absorb the GST-tagged protein in the resin. Subsequently, 1 mM ATP/MgSO_4_·7H_2_O was added to the mixture, which was shaken gently for 30 min at 37°C. To remove the supernatant, the mixture was centrifuged (5,800 × *g* for 2 min at 4°C). The residual resin binding to the GST-tagged protein was washed 15 times using 1 mL of PBS-T containing 1 mM ATP/MgSO_4_·7H_2_O. The GST-tagged protein was eluted five times in 1 mL of GST elution buffer (50 mM Tris-HCl [pH 8.0] and 10 mM reduced glutathione). Thereafter, the proteins were concentrated using a Vivaspin 500 concentrator with an MWCO of 50 kDa (Sartorius, Göttingen, Germany) or 30 kDa.

### Enzyme assays.

In the assays of ME and MDH activities for the oxidative reaction, NAD(P)H production was monitored as an increase in absorbance at 340 nm using a Shimadzu UV-1850 spectrometer (Shimadzu, Kyoto, Japan). The oxidative reaction catalyzed by ME was conducted in a 1-mL assay solution [100 mM Tris-HCl (pH 7 to 9), various concentrations of l-malate, various concentrations of NAD(P)^+^, various concentrations of NH_4_Cl, various concentrations of MnCl_2_, and 5 to 50 pmol enzymes]. The oxidative reaction catalyzed by MDH was conducted in a 1-mL assay solution [100 mM Tris-HCl (pH 7.0 to 9.0) or *N*-cyclohexyl-3-aminopropanesulfonic acid (CAPS)–NaOH (pH 9.5 to 11.0), various concentrations of l-malate, various concentrations of NAD(P)^+^, and 30 to 250 pmol enzymes]. After incubating the assay solution without l-malate at various temperatures for 5 min, l-malate was added to the solution to start the reaction. In the assays of ME and MDH activities for the reductive reaction, NAD(P)H consumption was monitored as a decrease in absorbance at 340 nm. The reductive reaction catalyzed by ME was conducted in a 1-mL assay solution (100 mM Tris-HCl, various concentrations of sodium pyruvate, various concentrations of NADPH, 50 mM KHCO_3_, 100 mM NH_4_Cl, various concentrations of MnCl_2_, and 25 to 200 pmol enzymes). The reductive reaction catalyzed by MDH was conducted in a 1-mL assay solution (100 mM Tris-HCl, various concentrations of oxaloacetate, various concentrations of NADH, and 10 to 100 pmol enzymes). After incubation of the assay solution without pyruvate or oxaloacetate at the optimum temperature for each enzyme for 5 min, pyruvate or oxaloacetate was added to the solution to start the reaction. One unit of enzymatic activity was defined as the amount of enzyme that could convert 1 μmol of substrate per minute.

### Calculation of kinetic parameters.

Except for *k*_cat_, the kinetic parameters were calculated by curve fitting based on the Hill equation (below) (48) using Kaleida Graph ver. 4.5.

$$v=V_{\text{max}}{\left[\text{S}\right]}^{n}{}^{\text{H}}\;/\left({\left[\text{S}\right]}^{n}{}^{\text{H}}+S_{0.\text{5}}{{}^{n}}^{\text{H}}\right)$$

The *k*_cat_ values were calculated by dividing the maximum reaction velocity (*V*_max_) by the molar number of enzymes.

### Coupled activity assays with *Sy*Fum.

The composition of a 1-mL assay solution when using *Sy*ME as a malate oxidation enzyme was as follows: 100 mM Tris-HCl (optimum pH of *Sy*Fum, pH 7.5) (20), 0.163 mM fumarate, 0.614 mM NADP^+^, 100 mM NH_4_Cl, 0.5 mM MnCl_2_, 100 pmol *Sy*Fum, and 100 pmol *Sy*ME. The composition of a 1-mL assay solution when using *Sy*MDH as a malate oxidation enzyme was as follows: 100 mM Tris-HCl (pH 7.5), 0.163 mM fumarate, 0.514 mM NAD^+^, 100 pmol *Sy*Fum, and 100 pmol *Sy*MDH. The concentrations of these substrates and coenzymes were the ratio of intracellular concentrations in *Synechocystis* 6803 (29). After starting the reaction at room temperature (23°C) by adding fumarate, NAD(P)H production was monitored as an increase in absorbance at 340 nm for 10 min using a Shimadzu UV-1850 spectrometer.

### Construction of vectors that were transformed into *Synechocystis* 6803 cells.

The genomic region containing the *me* gene (slr0721) with N-terminal EcoRI and C-terminal XhoI sites was synthesized and cloned into the pEX-A2J1 vector containing the Amp promoter by Eurofin Genomics Japan. The genomic region containing the *citH* gene (sll0891) with N-terminal BamHI and C-terminal XhoI sites was synthesized and cloned into the pEX-A2J1 vector by Eurofin Genomics Japan. Regarding the vector used for the construction of the ME-Comp strain, 500 bp upstream and 100 bp downstream of the *me* gene were introduced into N-terminal NdeI and C-terminal HpaI sites, respectively, and the region was cloned into the pTKP2031V vector by Eurofin Genomics Japan.

### Cyanobacterial strains and culture conditions.

A GT strain of *Synechocystis* 6803 isolated in 1988 (49) was used as the wild-type *Synechocystis* 6803 strain. The GT strain was cultivated in 70 mL of modified BG-11 medium consisting of BG-11_0_ liquid medium (50) buffered with 20 mM HEPES-KOH (pH 7.8). Both the ΔME and ΔMDH strains were cultivated in 70 mL of the modified BG-11 medium containing 0.3 μg/mL of chloramphenicol. The ME-Comp strain was cultivated in 70 mL of the modified BG-11 medium containing 0.8 μg/mL of kanamycin. The cultivation was conducted at 30°C under continuous white light (50 μmol photons/[m^−2^ · s^−1^]) with the cultures bubbled with 1% (vol/vol) CO_2_ in the air. The cell densities were calculated as optical density at 730 nm (OD_730_) using a Shimadzu UV-2700 spectrophotometer (Shimadzu, Kyoto, Japan), and the OD_730_ at the start of cultivation was fixed at 0.4.

### Immunoblotting.

Antibodies against *Sy*ME and *Sy*MDH were purchased from Cosmo Bio Co., Ltd. (Tokyo, Japan). Immunoblotting was conducted using 12% SDS-PAGE gels as described previously (51).

### Extraction of malate from *Synechocystis* 6803 cells.

After 3 days of cultivation, *Synechocystis* 6803 cells (OD_730_ × culture volume [mL] = 150) were collected by centrifugation (5,800 × *g* for 2 min at 25°C). The cells were suspended in 600 μL of 60% (vol/vol) methanol, and the suspension was mixed using a Twin Mixer TM-282 (Asone, Osaka, Japan) for 15 min. The suspension was centrifuged (20,400 × *g* for 5 min at 4°C), and 500 μL of the supernatant was collected. The supernatant was added to an Amicon Ultra 3-kDa-cutoff filter (Merck, Billerica, MA, USA) and centrifuged (20,400 × *g* for 60 min at 4°C). After centrifugation, 350 μL of the filtrate was dried using a CVE-2000 centrifugal evaporator (Eyela, Tokyo, Japan). The residue was dissolved in 30 μL of 100 mM Tris-HCl buffer (pH 8.0). The concentration of malate in the sample was measured using an E-kit for liquid l-malate (J. K. International, Tokyo, Japan).
