# Supplementary material for: Malic Enzyme, not Malate Dehydrogenase, Mainly Oxidizes Malate That Originates from the Tricarboxylic Acid Cycle in Cyanobacteria
Source: mBio. 2022 Oct 31;13(6):e02187-22. doi: 10.1128/mbio.02187-22 (PMC9765476; doi:10.1128/mbio.02187-22)
Supplement: TABLE S1 [file mbio.02187-22-s0001.docx]

**Table S1.**

| Name | Reaction | Biochemical analysis |
| --- | --- | --- |
| Citrate synthase | oxaloacetate + acetyl-CoA + H_2_O → citrate + CoA-SH | Ito et al. 2019 |
| Aconitase | citrate ↔ *cis*-Aconitate + H_2_O ↔ isocitrate | Nishii et al. 2021 |
| Isocitrate dehydrogenase | isocitrate + NADP^+^ → 2-oxoglutarate + NADPH + CO_2_ | Muro-Pastor and Florencio. 1992 |
| 2-Oxoglutarate decarboxylase | 2-oxoglutarate → succinic semialdehyde + CO_2_ | Wang et al. 2017 |
| Succinic semialdehyde dehydrogenase | succinic semialdehyde + NADP^+^ + H_2_O → succinate + NADPH | Ito and Osanai. 2020 |
| Succinate dehydrogenase | succinate + a quinone ↔ fumarate + a quinol | Not performed |
| Fumarase | fumarate + H_2_O ↔ malate | Katayama et al. 2019 |
| Malate dehydrogenase | malate + NAD^+^ ↔ oxaloacetate + NADH | Takeya et al. 2018 |
