# Supplementary figures and images for: Malic Enzyme, not Malate Dehydrogenase, Mainly Oxidizes Malate That Originates from the Tricarboxylic Acid Cycle in Cyanobacteria
Source: mBio. 2022 Oct 31;13(6):e02187-22. doi: 10.1128/mbio.02187-22 (PMC9765476; doi:10.1128/mbio.02187-22)

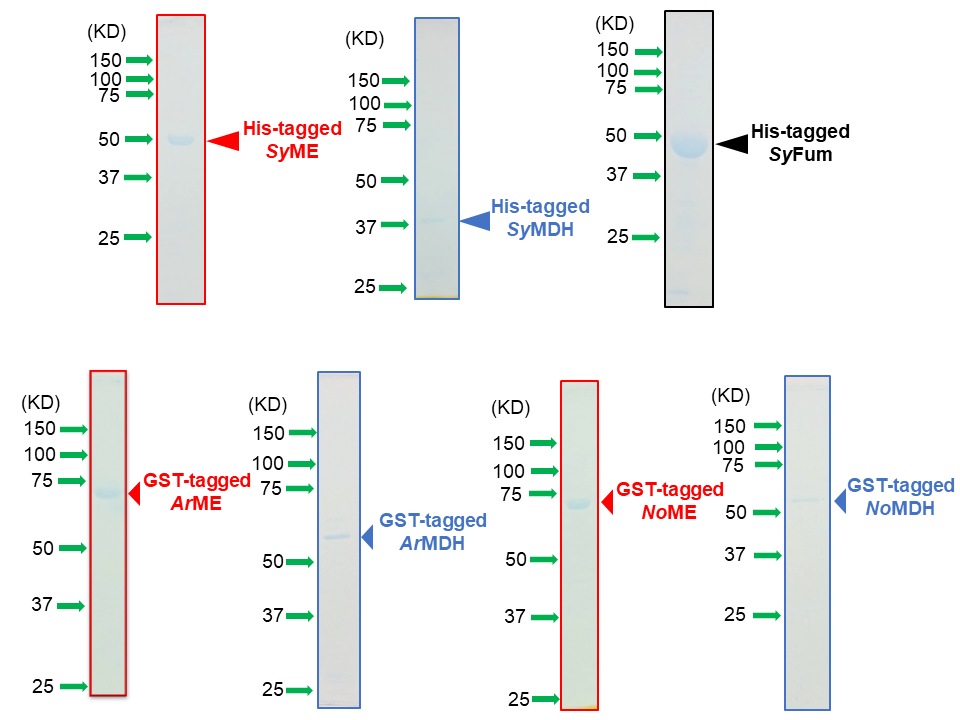

Supplement: FIG S1 [file mbio.02187-22-s0004.tif]

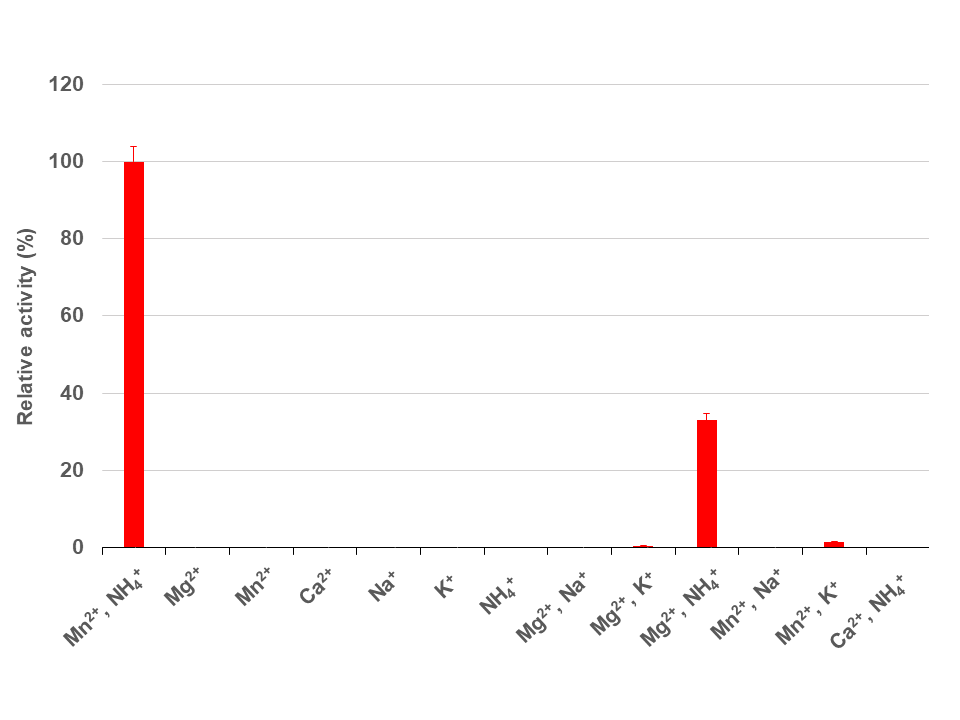

Supplement: FIG S2 [file mbio.02187-22-s0005.tif]

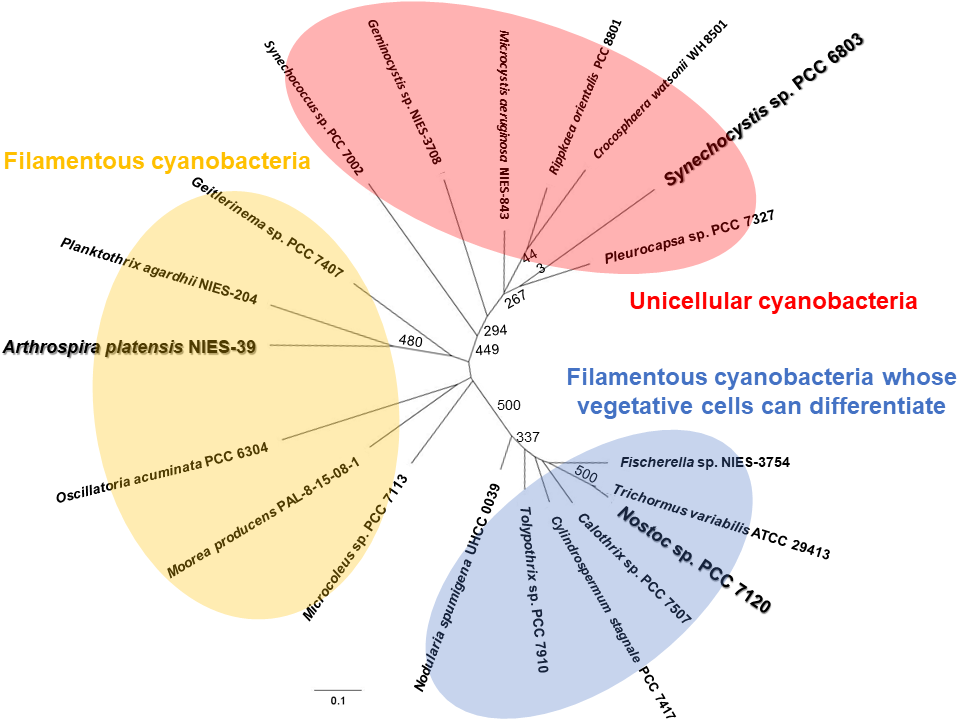

Supplement: FIG S3 [file mbio.02187-22-s0006.tif]

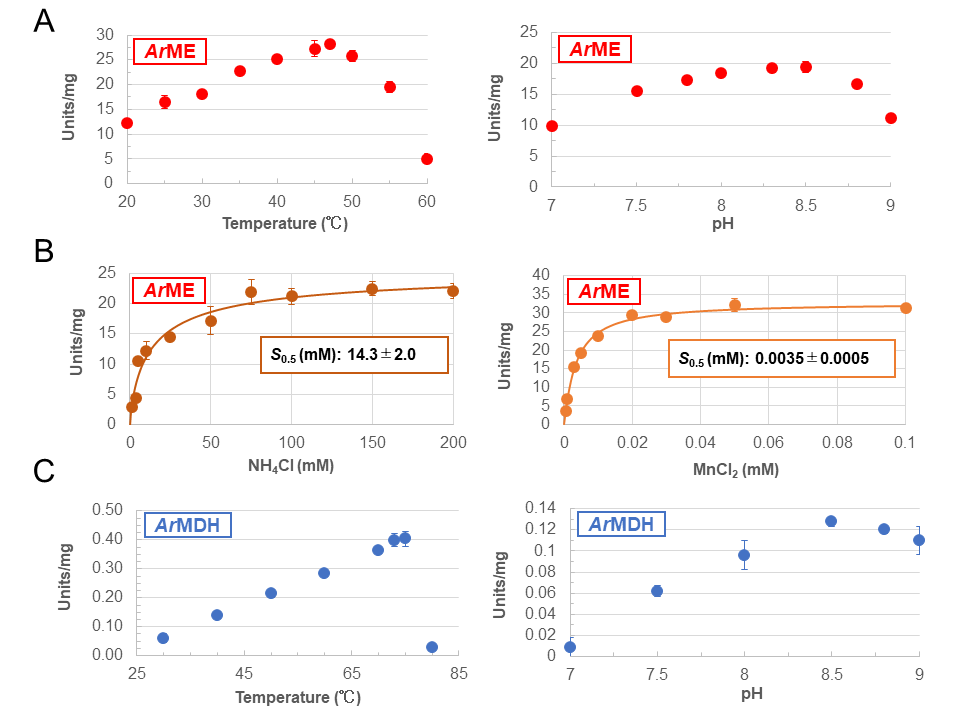

Supplement: FIG S4 [file mbio.02187-22-s0007.tif]

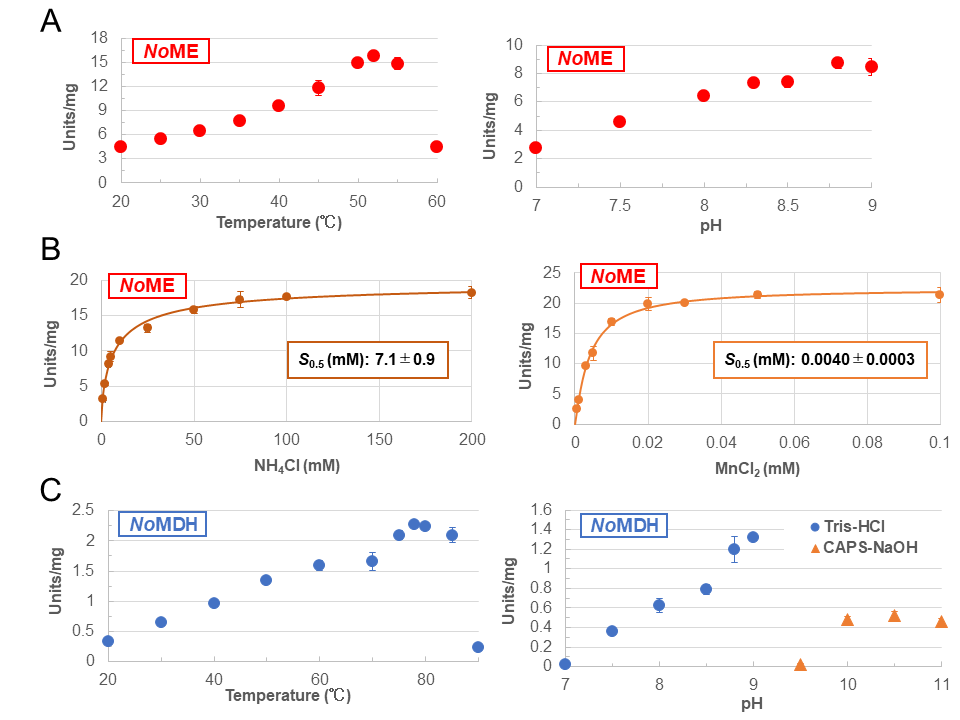

Supplement: FIG S5 [file mbio.02187-22-s0008.tif]

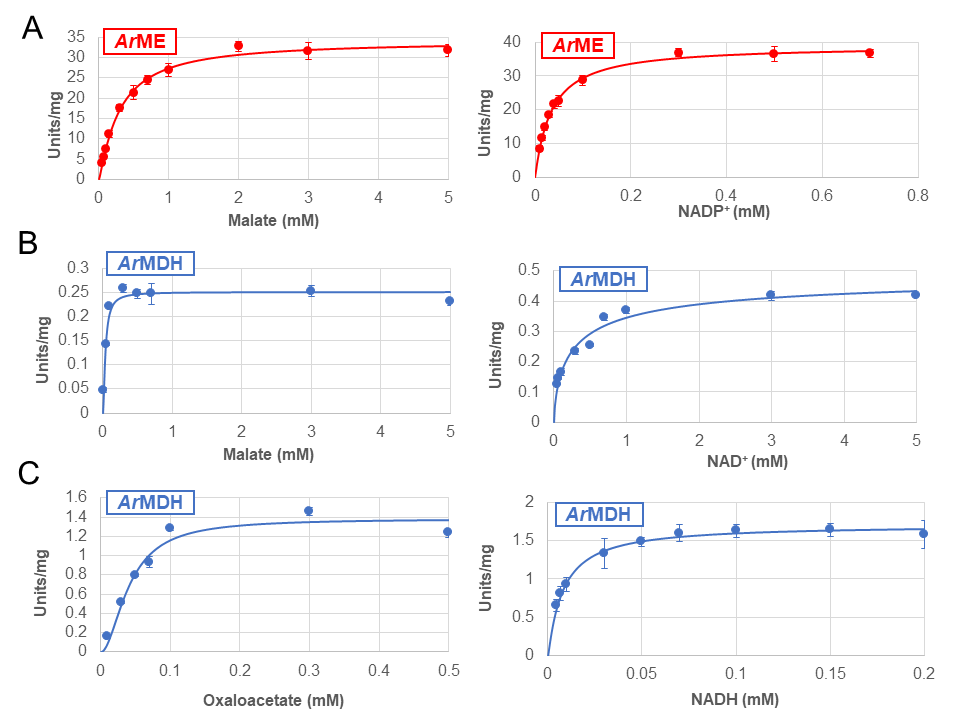

Supplement: FIG S6 [file mbio.02187-22-s0009.tif]

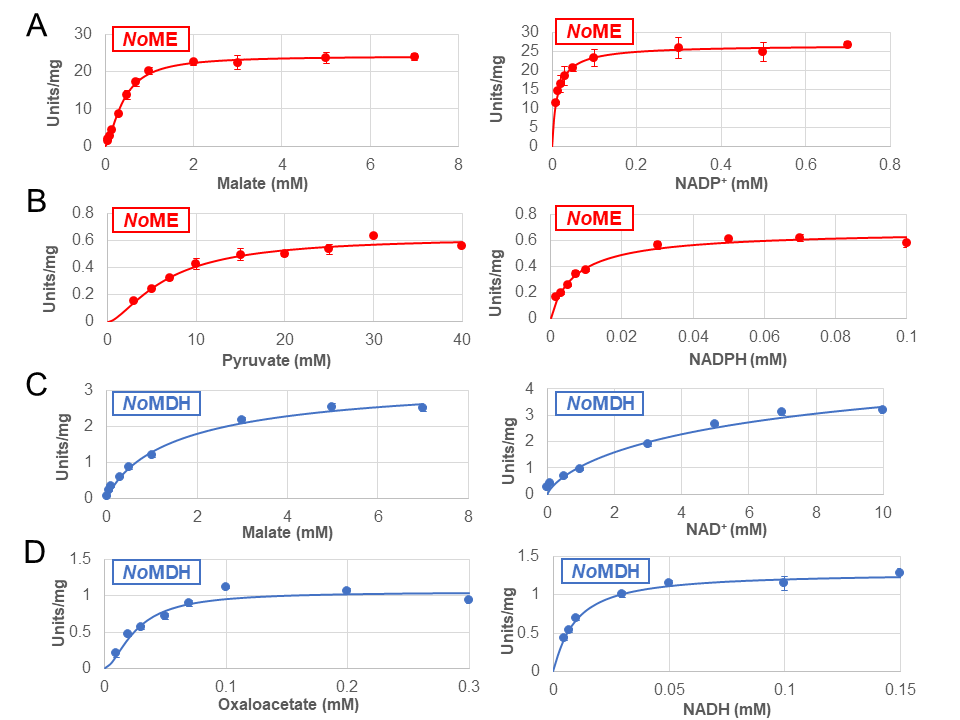

Supplement: FIG S7 [file mbio.02187-22-s0010.tif]
