# Supplementary material for: Malic Enzyme, not Malate Dehydrogenase, Mainly Oxidizes Malate That Originates from the Tricarboxylic Acid Cycle in Cyanobacteria
Source: mBio. 2022 Oct 31;13(6):e02187-22. doi: 10.1128/mbio.02187-22 (PMC9765476; doi:10.1128/mbio.02187-22)
Supplement: TABLE S2 [file mbio.02187-22-s0002.docx]

**Table S2.**

| Enzyme | *S*_0.5_ (mM) | *k*_cat_ (s^-1^) | *k*_cat_/*S*_0.5_ (s^-1^ mM^-1^) | *n*_H_ |
| --- | --- | --- | --- | --- |
| *Sy*ME | 4.89 ± 1.37* | 40.0 ± 5.4* | 8.4 ± 1.4** | 1.45 ± 0.24* |
| *Ar*ME | 6.55 ± 0.61** | 15.5 ± 0.9** | 2.4 ± 0.1** | 1.46 ± 0.10* |
| *No*ME | 5.43 ± 1.18* | 20.5 ± 1.29* | 3.9 ± 0.7* | 1.45 ± 0.14 |
